# Case Report: Osteosynthesis-associated infection with *Ochrobactrum intermedium* after acetabular fracture

**DOI:** 10.3389/fsurg.2024.1382564

**Published:** 2024-10-25

**Authors:** David Richard Krueger, Karl-Dieter Heller, Andrej Trampuz, Stefan Weenders

**Affiliations:** ^1^ Clinic for Orthopedic Surgery, Herzogin Elisabeth Hospital, Braunschweig, Germany; ^2^ Center for Musculoskeletal Surgery (CMSC), Charité - Universitätsmedizin Berlin, Berlin, Germany

**Keywords:** *Ochrobactrum*, *Ochrobactrum intermedium*, osteosynthesis-associated infection, acetabular fracture, hip arthroplasty

## Abstract

*Ochrobactrum intermedium* (*O. intermedium*) is a gram-negative, non-fermenting bacterium closely related to *Brucella* genus. *O. intermedium* resembles an emergent human pathogen that has rarely been detected in both immunocompetent and immunodeficient patients. A musculoskeletal infection with *O. intermedium* has not been described in the literature. We present the first case of an osteosynthesis-associated infection (OAI) with *O. intermedium* in an 80-year-old female patient after osteosynthesis of an acetabular fracture. The patient was admitted to the emergency department 6 months after osteosynthesis of a posterior column acetabular fracture treated via open reduction and internal plate fixation of the posterior column. The patient demonstrated tenderness, redness and swelling at the insertion site as well as a fistula. The radiological controls showed femoral head necrosis and partial protrusion of the head into the pelvis. The laboratory parameters showed no pathological findings. OAI was assumed and a two-stage revision with implant removal and resection arthroplasty in the first stage and hip arthroplasty in the second stage was performed. All microbiological specimens taken at the osteosynthesis site and the hip joint grew *O. intermedium*. The pathogen was determined using the matrix-assisted laser desorption/ionization time-of-flight (MALDI-TOF) method. Antibiotic regime consisted of intravenous (IV) meropenem for two weeks followed by oral ciprofloxacin and cotrimoxazole. Implantation of the hip prosthesis was performed 6 weeks after the index surgery using a cementless revision cup and a cemented stem. Meropenem and vancomycin IV were given for one week followed by ciprofloxacin and doxycycline for another 5 weeks. 24 months after the surgery, the patient is infection free and satisfied with the result. With this case report we would like to increase awareness of possible implant-associated bacterial infections caused by *O. intermedium*.

## Introduction

*Ochrobactrum intermedium* (*O. intermedium*) is a gram-negative, non-fermenting bacterium closely related to *Brucella* genus (1). *O. intermedium* resembles a nascent human pathogen that has rarely been detected so far (1–8). To date, there are only 8 case reports of an infection with *O. intermedium* (1–8). They are found in immunocompromised and immunocompetent patients and the clinical presentation ranges from severe bacteraemia to incidental findings (1–8).

A musculoskeletal infection with *O. intermedium* has not been reported in the literature. We present the first case of an osteosynthesis-associated infection (OAI) with *O. intermedium* in a 80-year-old female patient after osteosynthesis of a posterior column acetabular fracture type 62A2.2 according to the AO classification via a Kocher-Langenbeck approach.

## Case description

**Figure 1:**
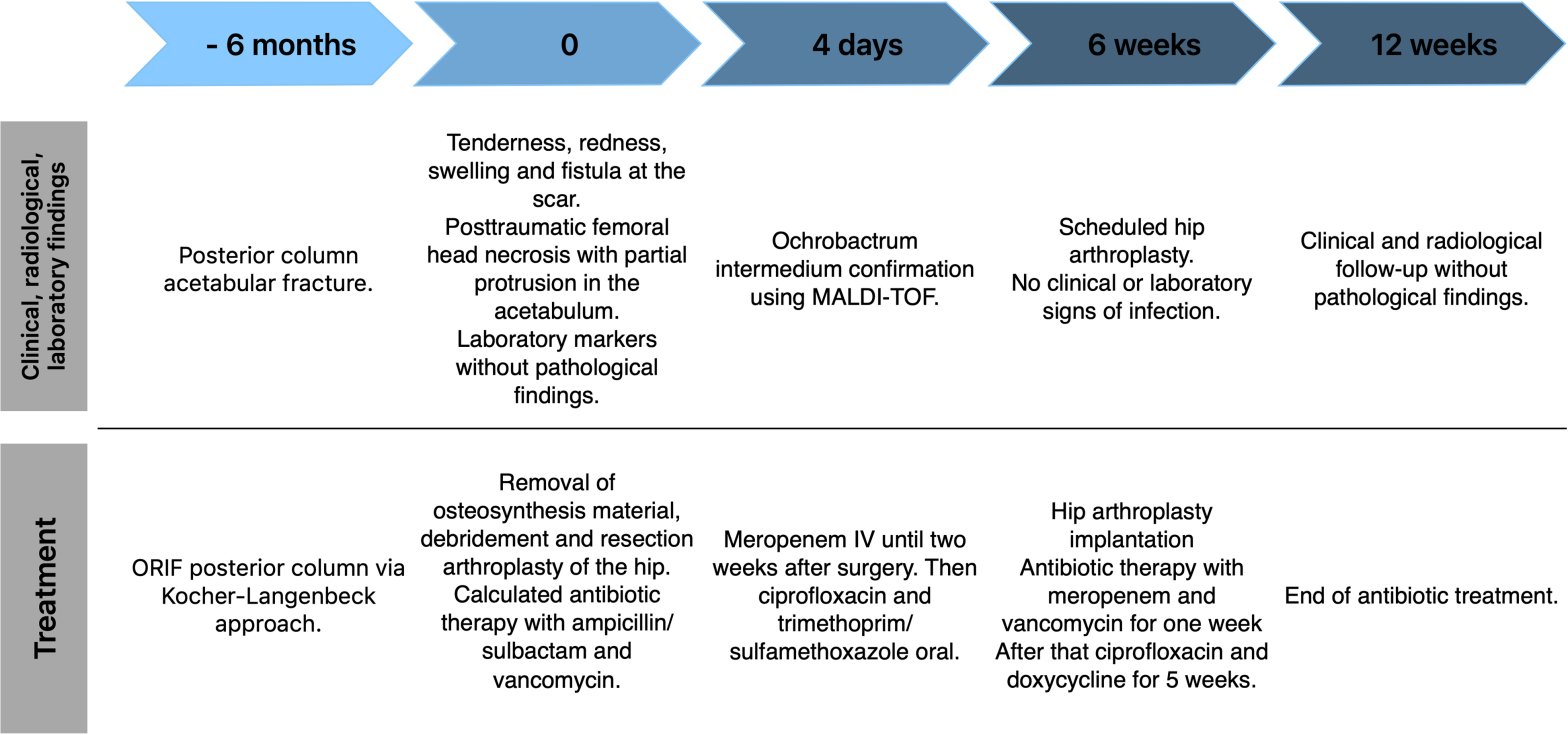
Case report timeline.

**Figure 2:**
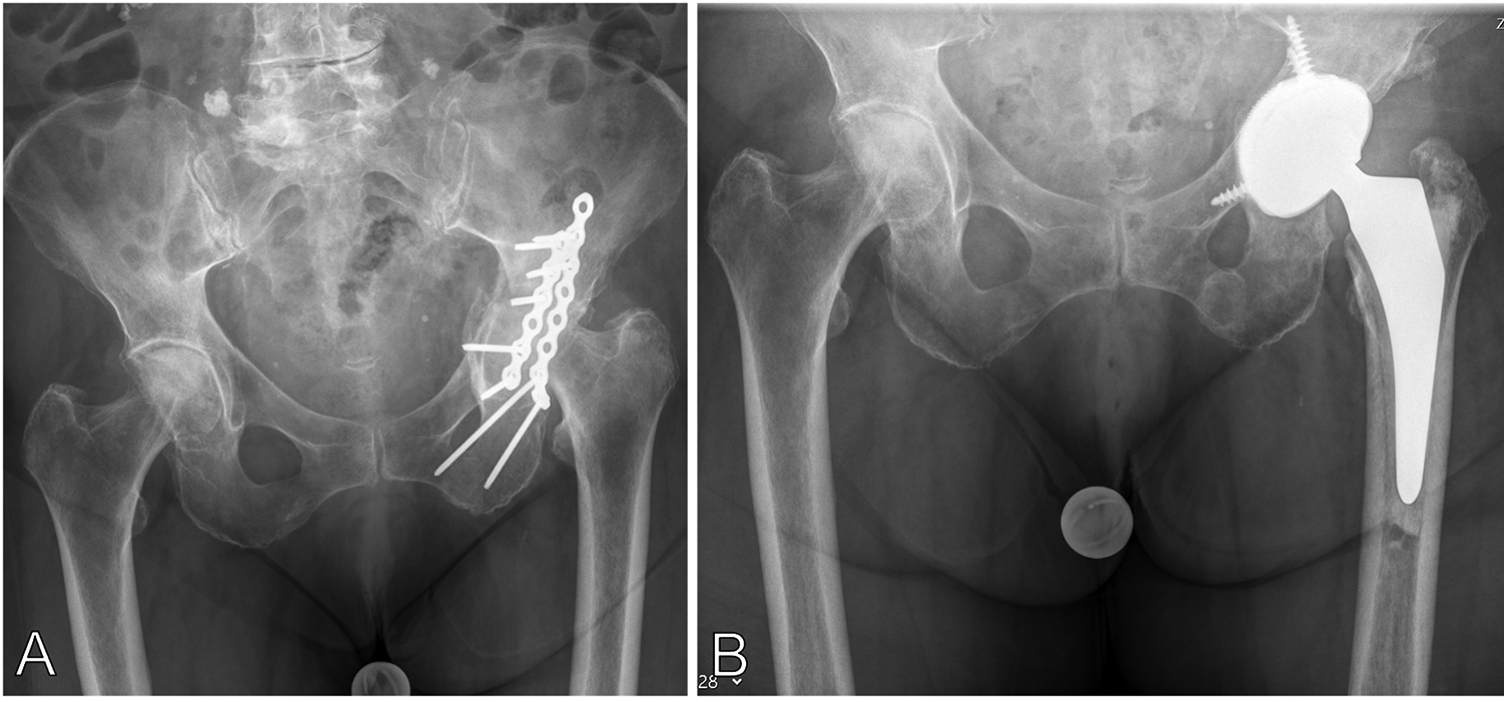
**(A)** AP pelvis x-ray showing partial femoral head necrosis and acetabular protrusion after open reduction and internal plate fixation of a posterior column acetabular fracture via a Kocher-Langenbeck approach. **(B)** Postoperative anterior posterior (AP) Pelvis 6 weeks after implantation of a hybrid cemented total hip arthroplasty using a cementless revision cup with additional screw fixation and a cemented stem.

The historical and current information from this episode of care is organized as a timeline in Figure 1. An 80-year-old female was admitted to the emergency department with pain in her left hip. Six months previously she had undergone open reduction and internal plate fixation of the acetabular fracture via a Kocher-Langenbeck approach. At the time of osteosynthesis, there was no indication of immune deficiency. The patient had no history of frequent infections or infections with opportunistic pathogens. The serological infection parameters were normal at the time of osteosynthesis (Leukocytes 10,870/µl, C-Reactive Protein 2.5 mg/L). The postoperative period was without complications and primary wound healing occurred. The patient demonstrated tenderness, redness and swelling at the scar as well as a fistula. The x-ray showed posttraumatic femoral head necrosis with partial protrusion (Figure 2). Two of the screws communicated with the hip joint as confirmed by computed tomography. Laboratory markers were without pathological findings regarding infection (Leukocytes 6,450/µl, C-Reactive Protein 3.6 mg/L). The patient's medical history showed hypertension, hyperlipidaemia and a cholecystectomy. Her Body Mass Index (BMI) was 24 kg/m^2^. The patient had a history of anterior pelvic ring injury with fracture of the left inferior and superior pubic ramus five years ago, which was treated conservatively.

Due to the fistula and the clinical presentation, OAI was assumed. Because of the communication of the osteosynthesis material with the hip joint and the femoral head necrosis, an infection of the hip joint was expected as well. A two-stage revision was planned. In the first stage, removal of the osteosynthesis material, debridement and resection arthroplasty of the hip joint with removal of the femoral head (Girdlestone procedure) was performed. Due to the risk of protrusion into the pelvis following the acetabular fracture, we did not use a spacer. Calculated postoperative antibiotic therapy was performed with ampicillin/sulbactam and vancomycin. All of the seven intraoperative microbiological cultures from the site of the osteosynthesis and from the hip joint yielded *O. intermedium*. The pathogen was determined using the matrix-assisted laser desorption/ionization time-of-flight (MALDI-TOF) method. The antibiotic susceptibility profile is displayed in Table 1.

**Table 1:** Antibiotics susceptibility table for *Ochrobactrum intermedium*.

Antibiotics	Susceptibility profile
Piperacillin	Resistant
Piperacillin/Tazobactam	Resistant
Cefotaxim	Resistant
Cefepim	Resistant
Ceftazidim	Resistant
Imipenem	Sensitive
Meropenem	Sensitive
Aztreonam	Resistant
Ciprofloxacin	Sensitive
Moxifloxacin	Resistant

After detection of the pathogen, the antibiotic therapy was changed to meropenem 3 × 1 g IV. After IV therapy for two weeks, the therapy was changed to oral antibiotics with ciprofloxacin 2 × 750 mg and cotrimoxazole 3 × 960 mg. The antimicrobial therapy was planned in consultation with an infectious disease specialist according to the Pro-Implant Foundation criteria.

Mobilization was performed with minimal weight bearing on crutches until the final hip arthroplasty implantation. The inpatient stay was 14 days and without complications. Wound healing was normal. The laboratory infection parameters, which increased after the operation and continuously decreased until discharge.

Six weeks after debridement and resection arthroplasty, the scar showed no signs of infection and the inflammatory biomarkers were in the normal range (Leukocytes 6,010/µl, C-Reactive Protein 1.6 mg/L). Implantation of the hip arthroplasty was performed via the same posterolateral approach using a cementless revision cup with screw fixation (Plasmafit® Revision, Aesculap, Tuttlingen, Germany) for the acetabulum and a cemented standard stem (CoreHip®, Aesculap, Tuttlingen, Germany). The postoperative antibiotic therapy consisted of meropenem 3 × 1 g and vancomycin 2 × 1 g IV for one week. After that, an oral antibiotic therapy with ciprofloxacin 2 × 750 mg and doxycycline 2 × 100 mg was started and continued for another 5 weeks. Application of local antibiotics was not performed in this case. After hip arthroplasty the wound healing proceeded normally and the infection parameters decreased until discharge. After implantation of the hip arthroplasty, the patient was allowed full weight bearing.

At the 6-week follow-up the patient showed no pain and no clinical or laboratory signs of infection. Radiological control showed a normal position of the hip arthroplasty without signs of loosening or periprosthetic fracture (Figure 2).

24 Months after implantation of the hip arthroplasty the patient is free of infection and satisfied with the result.

## Discussion

*O. intermedium* is a gram-negative, non-fermenting organism and represents an emerging species that was first separated from *O. anthropi* in 1998 since standard cultivation and tests did not allow an exact differentiation (9). Until then the non-fermenting bacterium *Ochrobactrum anthropi* has been the only species of the *Ochrobactrum* genus (9).

Sharing nearly 99% of its 16S rRNA, *O. intermedium* has a closer relation to the Brucella genus than other species of *Ochrobactrum*. Differentiation is only possible with advanced molecular tests like 16S rDNA gene sequencing recA-PCR RFLP (Restriction Fragment Length Polymorphism), and the MALDI-TOF method (9). In our case *O. intermedium* was specified using the MALDI-TOF method.

So far, the non-fermenting bacterium *Ochrobactrum anthropi* has been the only species of the genus (9). *Ochrobactrum*, a common environmental pathogen found in soil and water, is closely related to *Brucella spp.* due to their similar genetic and physiological characteristics (10).

*Ochrobactrum anthropic* could be isolated in various clinical specimens and seems to be part of the flora of the large intestine (11). In contrast to O. anthropic, only a few cases of *O. intermedium* infections have been described in the literature. Möller et al. presented the first case of an infection with *O. intermedium* in 1999 in a liver transplanted patient with bacteraemia and liver abscesses due to *O. intermedium* (1). Other case reports include immunocompromised patients with bowel obstruction and bladder cancer as well as a case of endocarditis and bacteraemia after cholangitis (2–4). Other case reports present immunocompetent patients with appendicitis complicated by a perirectal abscess and a catheter-associated infection after a stroke (5, 6). In one case *O. intermedium* was found incidentally (7). In our case the patient's comorbidities were limited to hypertension, hyperlipidaemia and a cholecystectomy many years ago. There was no history of frequent or opportunistic infections or other chronic diseases. Malnutrition might have played a role in the patient with a BMI of 24 kg/m^2^, but since it was only slightly below 25 kg/m^2^, we did not rate this factor to be clinically relevant. We consider the patient to be immunocompetent.

This is the first case to present an implant related infection with *O. intermedium*.

A limitation of this case report that should be acknowledged is the mid-term follow-up period of 24 months, so far. We will continue to follow the patient after hip arthroplasty implantation.

Identification of the pathogen in an osteosynthesis-associated infection or a periprosthetic joint infection is crucial in order to be able to initiate targeted therapy. Culture negative infection is associated with worse treatment success in periprosthetic joint infections (12). The challenge lies in the identification of *O. intermedium* and highlights the potential of advanced molecular tests like 16S rDNA gene sequencing recA-PCR RFLP and the MALDI-TOF method.

## Conclusion

Infection with *O. intermedium* is rarely identified due to the need of advanced molecular tests for its differenciation. Still *O. intermedium* has been recognized as pathogen seen in immunodeficient and immunocompetent patients. We described the first case of an osteosynthesis-associated infection with *O. intermedium* in an 80-year-old patient after osteosynthesis for acetabular fracture. With this case report we would like to increase awareness of possible implant-associated bacterial infections caused by *O. intermedium*. Two-stage hip arthroplasty with implant removal, debridement and resection arthroplasty in the first stage and hip arthroplasty implantation in the second stage with targeted antibiotic therapy led to a good short-term result in this patient.

## Data Availability

The original contributions presented in the study are included in the article/Supplementary Material, further inquiries can be directed to the corresponding author.
